# Eleventh Annual Meeting of American Society of Dental Surgeons

**Published:** 1850-10

**Authors:** C. O. Cone

**Affiliations:** Cor. and Rec. Secretary.


### AMERICAN SOCIETY OF DENTAL SURGEONS.

### ELEVENTH ANNUAL MEETING OF THE AMERICAN SOCIETY OF DENTAL SURGEONS.

The eleventh annual meeting of the American Society of Dental Surgeons, was held, August, 1850, at Saratoga Springs.

The Society convened at Union Hall, on Tuesday, August 13th, 1850, at 12 M., Dr. E. Townsend, Vice-president, in the chair.

Members of the Society present were as follows: namely, Drs. E. Townsend, C. A. Harris, J. H. Foster, E. Noyes, L. S. Parmly, Jahial Parmly, D. R. Parmly, A. Hill, W. H. Dwinelle, Jas. Alcock, Wm. Dalrymple, E. J. Dunning, H. H. Young, R. Nelson, J. B. Rich, A. C. Hawes, T. Palmer and C. O. Cone.

On motion of C. A. Harris, C. O. Cone was elected Secretary pro tem.

On motion, C. O. Cone, A. Hill and L. S. Parmly were selected a committee of three to arrange the business of the Society. The Society then adjourned to meet at 4 o'clock, P. M.

*4 o'clock, P. M.*

The Society met agreeably to adjournment.

The report of the committee for arranging the business of the meeting was made and adopted by the association.

The committee of investigation being the first in order, on being called, reported progress.

The report of the tabular sheet, of statistical facts was made by Dr. Cone, and adopted.

The committee on Amalgam appointed at the last meeting of the Society then presented their report through their chairman, Dr. Townsend, and which, on motion of Dr. L. S. Parmly, was adopted. (See page 66.)

After a debate, on motion made by Dr. A. Hill to adopt the resolution accompanying the report of the committee, in which Drs. Rich, J. Parmly, D. R. Parmly, C. A. Harris, J. H. Foster, A. Hill and W. H. Dwinelle, took part; Dr. Hill withdrew his motion for the adoption of the report. The Society then on motion of Dr. Dwinelle, rescinded its vote accepting the report of the committee on amalgam, and on motion of Dr. Dwinelle, referred the report back to said committee, with power to revise or amend.

The committee on amalgam after a short consultation, presented their report amended, which was adopted.

The Society then adjourned to meet at 8 o'clock, P. M.

*8 o'clock, P. M.*

The Society met agreeably to adjournment.

On motion of Dr. C. A. Harris, the resolution accompanying the report of the committee on amalgam appointed by the last meeting of the Society, and which reads as follows, namely:

***Resolved,*** That the several resolutions adopted by the American Society of Dental Surgeons at the annual meeting held 1845, and 1846, having the effect of enforcing subscription to the protest and pledge against the use of amalgams and mineral paste fillings for teeth, be, and the same are hereby rescinded and repealed—was unanimously adopted.

Dr. J. B. Rich then tendered his resignation, which was accepted by the Society.

The Society then adjourned to meet at 9 o'clock, on Wednesday morning.

*Wednesday, 9 o'clock, A. M.*

The Society met agreeable to adjournment.

The proceedings of the previous day having been read and approved. Dr. C. A. Harris, on behalf of the committee on the publication of the Journal, its present condition, and its future publication; made a verbal report, which was accepted.

The following resolutions were then offered by Dr. A. Hill, and adopted by the Society, namely:

***Resolved,*** That the future publication of the American Journal and Library of Dental Science be, and hereby is discontinued by this Society, and all future ownership of the Society in the publication of the said Journal be surrendered to such member or members of this Society as shall assume itsdebts, giving security for the same to the President of this Society, or his successor in office.

***And be it further Resolved,*** That the President of this Society be, and he is hereby authorized to make the necessary transfer of said Journal to Dr. C. A. Harris, agreeably with the foregoing resolution.

On motion of Dr. C. O. Cone, the following resolutions were adopted:

***Resolved,*** That so much of the 1st section of the third article of the Constitution of this Society, which relates to creating two distinct offices or secretaryships, be amended, so that the first section of the third article of the Constitution shall read as follows: namely,

ARTICLE III.

Section 1. The officers of the Society shall consist of a President, three Vice-presidents, a Corresponding and Recording Secretary, a Treasurer, a Librarian, an Executive Committee, consisting of seven members, and a Publishing Committee, consisting of three members.

***And be it further Resolved,*** That so much of the by-laws of this Society, as relates to the duty of two distinct secretaryships shall be embraced in one.

***And be it further Resolved,*** That so much of the By-laws of this Society as relates to the emolument of the Corresponding Secretary, shall be and the same is hereby rescinded and repealed.

The Society then went into an election of officers for the ensuing year which resulted as follows:

| | |
|---|---|
| ELEAZER PARMLY, of New York, | PRESIDENT. |
| ELISHA TOWNSEND, of Philadelphia, | 1ST VICE-PREST. |
| J. H. FOSTER, of New York, | 2ND " |
| J. A. CLEVELAND, of Charleston, | 3D " |
| C. O. CONE, of Baltimore, | COR. & REC. SEC. |
| E. J. DUNNING, of New York, | TREASURER. |
| J. H. FOSTER, | LIBRARIAN. |

C. O. CONE,
E. J. DUNNING,
D. R. PARMLY, } PUBLISHING COMMITTEE.

J. H. FOSTER,
E. J. DUNNING,
E. PARMLY,
A. C. HAWES,
C. A. HARRIS,
H. N. FENN, } Executive Council and Examining Committee.

On motion of Dr. Harris,

***Resolved,*** That the word Council be substituted for the word Committee, in section 1st, article 3d of the Constitution, so that it shall read Executive Council and Examining Committee.

On motion of Dr. Cone,

***Resolved,*** That the number of the committee on Practical Dentistry be, and hereby is, reduced to one member.

Dr. E. Townsend was then appointed a committee to prepare for the next annual meeting, a report on Practical Dentistry.

On motion of Dr. C. A. Harris, the Society adopted the following resolutions:

***Resolved,*** That six members of the Society be appointed to complete the number of aphorisms called for by the Society some years ago, with a request that they prepare them immediately, and place them in the hands of the publishing committee; and,

***Resolved,*** furthermore, That the publishing committee revise and publish, as soon as practicahle, said aphorisms, and that at the expense of the Society, and furnish such publication to members of the Society at cost.

The following members of the Society were then selected to prepare the aphorisms, agreeably to the foregoing resolutions.

E. Townsend, E. J. Dunning, A. C. Hawes, J. H. Foster, and C. O. Cone.

On motion of Dr. J. H. Foster, a committee was appointed to take into consideration and report on the expediency of revising so much of the constitution as related to the admission of new members; Drs. J. H. Foster and Jahial Parmly were appointed that committee.

Dr. N. A. Fisher, of Providence, R. I., communicated to the Society, through Dr. A. C. Hawes, his intention of abandoning the practice of the profession, from ill health, and tendered his resignation, which was accepted.

The committee to whom was referred so much of the constitution, as relates to the admission of new members, reported, that in the opinion of the committee, it was inexpedient to make any revision or alteration of that portion of the constitution which was referred to them for their consideration.

The resignation of Charles Meritt, of Bridgeport, Conn., was then tendered, and accepted.

On motion of Dr. C. O. Cone,

*Resolved,* That all of the surplus numbers of the American Journal and Library of Dental Science, now published, and on hand, be equally divided between C. A. Harris, Amos Westcott and William H. Dwinelle, former editors of said periodical, after each member of this Society, now in good standing, shall have been supplied with such back numbers of those now on hand, as shall complete their sets from the time of their subscription, to the tenth volume, provided they shall have been and are, on the completion of the tenth volume, subscribers to the same.

The Society then adjourned to meet at 4 o'clock, P. M.

4 *o'clock, P. M.*

Society met agreeable to adjournment, Dr. Foster in the chair.

The committee of investigation, appointed on motion of Dr. Dwinelle, at the last meeting, reported that after a full examination of the case, they find no cause of action against the individual implicated, and that he stands fully vindicated before the Society, which report,

On motion of Dr. C. A. Harris the report was then adopted by the Society.

The Society then adjourned to meet on Thursday morning at 9 o'clock.

*Thursday Morning, Aug.* 15*th,* 1850.

The Society met agreeably to adjournment, Dr. Townsend in the chair; the proceedings of yesterday were read by the secretary, and approved.

The minutes of the last meeting of the Society, held in the city of Baltimore, the 25th, 26th and 27th of March, 1850, and as published in the American Journal of Dental Science, in the absence of the records of the Society, was then read by the secretary, agreeably to the order of the association.

On motion of Dr. C. A. Harris,

*Resolved,* That portion of the minutes of the last meeting of the association, published on page 209 of the tenth vol. of the American Journal and Library of Dental Science, which refers to a motion made by Dr. Cone, requesting a copy of memoir of the late Dr. Nasmyth, for publication, be, and is so corrected as to embrace some prefatory remarks made by Dr. Harris, accompanying the same.

On motion made by Dr. W. H. Dwinelle, the Society adopted the following resolution:

*Resolved*, That the minutes of the proceedings of the Society at its meeting in Baltimore, on the 25th, 26th and 27th days of March, 1850, as published in the American Journal and Library of Dental Science, at pages 208, 9 and 10, be, and the same are hereby corrected, by striking therefrom all such portions as relate to the formation or proceedings of what is there styled a committee on grievances; for the reason that all such portions may tend to throw an unwarrantable and unjustifiable suspicion on the character of one of the members of this Society. Such record being an error, as no charges was preferred, it having been regarded by all present that no minute of the case should be recorded.

Dr. Dwinelle then moved that a committee of investigation be appointed to report to the next meeting.*

On motion of Dr. Harris,

*Resolved*, That the committee on Dental Literature be continued to report at next meeting.

On motion of Dr. Harris, Dr. S. P. Hullihen was continued a com mittee to prepare an address for the public, and report at the next meeting.

On motion of Dr. Harris. the resolution of the Society accepting the resignation of Joshua and E. G. Tucker be, and hereby is, rescinded.

On motion of J. H. Foster,

*Resolved*, That the chairman of the executive council be, and hereby is allowed to withdraw Joshua and E. G. Tucker's resignation of membership of this association, and that they be, and hereby are, reinstated in full and active membership from this time.

Dr. Foster, chairman of the executive council, then reported to the association the names of E. D. Wheeler of Murfeesborough, Tenn., and J. D. White of Philadelphia as candidates for membership, they having furnished the vouchers prescribed by the constitution and by-laws, whereupon they were unanimously elected active members of the Society.

Dr. Foster then reported the name of N. A. Fisher as an honorary member, unanimously, elected.

*With regard to the error referred to, in the above resolution, in the published proceedings of the called meeting of the Society, held in March, it is due to the recording secretary to say, that when he sent the manuscript copy, he wrote a letter, requesting us to read it, and make such alterations and corrections as we might deem proper. But being sick at the time, and as the printer was waiting for copy, we sent the manuscript immediately to him, and entrusted to him, the reading of the proof. The letter entirely escaped our notice at the time, and it was several months before we saw it.—*Editor.*

Dr. L. S. Parmly, of New Orleans, then tendered his resignation, which was accepted by the Society.

On motion of Dr. C. A. Harris,

*Resolved,* That Dr. L. S. Parmly be, and hereby is elected an honorary member of this association.

On motion of Dr. C. O. Cone, Dr. J. H. Foster was elected to deliver the opening address at the next annual meeting of the Society.

The following members were then elected to deliver essays upon some subject connected with dental theory and practice, at the next annual meeting. Dr. E. G. Tucker, of Boston, Dr. A. Hill, of Norwalk, Ct., Dr. E. J. Dunning, of New York, Dr. A. C. Hawes, of Providence. R. I., Dr. R. Nelson, of Albany, New York.

On motion of Dr. C. A. Harris,

*Resolved,* That the Society when it adjourns, it shall adjourn to meet at the United States Hotel, in the city of Philadelphia, on the 1st Tuesday of August, 1851.

On motion of Dr. C. A. Harris,

*Resolved,* That Dr. E. J. Dunning be appointed a committee to receive all the papers, books and property of the Society, in the hands of the former secretary, Dr. A. Westcott, and transmit the same to the present secretary, elected for the year ensuing.

Dr. A. Hill then entertained the association with the history of a series of experiments he had been engaged in, and presented a number of metallic plates for mounting artificial teeth, obtained by the electrotype process, as the result of such experiments.

On motion of Dr. C. A. Harris,

*Resolved,* That the thanks of the association be tendered Dr. A. Hill, for making public the results of his experiments in obtaining, by the electrotype process, metallic plates for mounting artificial teeth, together with a request that he continue his experiments in the same, and report the result to the next meeting of the association.

On motion of Dr. C. A. Harris,

*Resolved,* That the American Society of Dental Surgeons, deeming the preservation of the health of the dental organism of the highest importance to the comfort and welfare of the whole human economy, approve of the system of hygiene recommended by Dr. L. S. Parmly.

Dr. Dwinelle then presented to the Society a set of artificial teeth mounted for the inferior jaw, showing a novel method of mounting the same, adopted by Dr. Geo. E. Hawes, of New York.

On motion of Dr. C. O. Cone, Dr. Hawes, was requested to detail to the Society, the method he adopted, to attain the artistical perfection in the construction of the piece just described.

On motion of Dr. C. A. Harris,

*Resolved,* That the thanks of the Society be tendered to Dr. George E.

Hawes for the explanation of a new method of mounting artificial teeth for the inferior jaw.

The subject of adopting metallic plates to the inferior jaw was discussed by Drs. Dwinelle and Dunning, with explanations by Dr. Dwinelle of a method adopted by him in difficult cases, and a description of casting gold plates for the inferior arch by Dr. Dunning. Dr. Cone referred to plans adopted by Dr. P. H. Austin, of Baltimore, for withdrawing difficult casts from sand, so as to secure a perfect mould. Dr. Hawes, by request of the Society, then explained a casting-box which he employed to attain the same end.

The Society then adjourned to meet on the first Tuesday of August, in the city of Philadelphia.

C. O. CONE,
Cor. and Rec. Secretary.

